# Visually augmented targeted combination light therapy for acne vulgaris: a case report

**DOI:** 10.1186/s13256-017-1469-y

**Published:** 2017-10-31

**Authors:** Alireza Yazdi, Colin-William Lyons, Niamh Roberts

**Affiliations:** 1 Department of Primary Care and Public Health, Faculty of Public Health, Imperial College London, London, UK; 2 Gordano School, Portishead Bristol, UK

**Keywords:** Acne vulgaris, Phototherapy, Light therapy, Dermatology

## Abstract

**Background:**

Acne vulgaris is a common skin disease. Pharmacological modalities for treatment are proven to be efficacious but have limitations. Light therapy for acne vulgaris has shown promise in previous studies. This case report and its accompanying images show how a novel approach of visually augmented high fluence light therapy has been used to good effect.

**Case presentation:**

A 26-year-old Caucasian woman with acne vulgaris resistant to treatment with topical therapy underwent three sessions of combination potassium titanyl phosphate laser (532 nm)/neodymium-doped: yttrium aluminum garnet laser (1064 nm) light therapy with visually augmented narrow spot size and high fluence. A 73% reduction in total inflammatory lesions was evident 6 months after the initial treatment.

**Conclusions:**

This case report illustrates that there may be utility in this novel approach of narrow spot size, magnification-assisted, high fluence targeted combination laser therapy for inflammatory acne.

## Background

Acne vulgaris is a common skin disease affecting 90% of people at some time in their lives, to varying degrees. It can have a profound psychological impact on the patient, often out of proportion from the perceived physical effect.

Established medical treatment for acne includes topical application of benzoyl peroxide, topical and oral antibiotics, and systemic retinoids for severe or resistant acne. Although proven to be efficacious, these treatments have disadvantages that limit their use. Systemic retinoids are teratogenic and have systemic side effects, while non-compliance is an issue with topical treatment. There is also the problem of growing antibiotic resistance of the *Cutibacterium acnes* organism that contributes to inflammation in acne [1].

There has been recent interest in the role of light therapy in acne. A recent Cochrane review concluded that high quality evidence was lacking, but that treatment with green light was more effective than placebo [2]. There have been a number of promising results from studies on a range of energy-based devices [3].

The following case illustrates an approach to using phototherapy for acne that uses a novel combination of narrow spot size wavelengths with high energy level phototherapy under visual magnification to achieve a 73% reduction in inflammatory lesions. A literature search of acne phototherapy indicates that this is a unique approach that merits further investigation.

## Case presentation

**Fig. 1:**
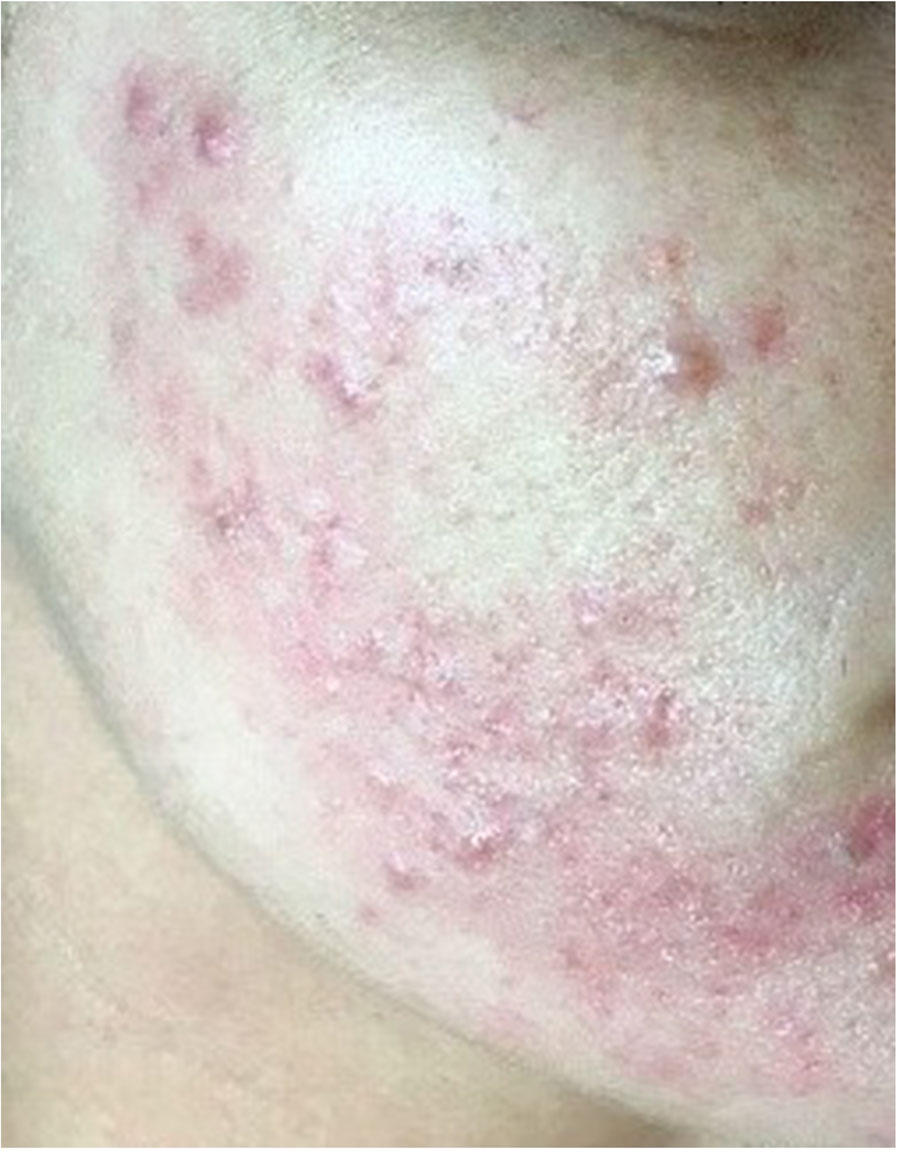
Baseline, prior to first treatment

A 26-year-old Caucasian woman with Fitzpatrick skin type II presented with facial acne vulgaris. She had a slim build, no evidence of hirsutism, and no significant past medical or family history. Multiple inflammatory lesions (37 in total), predominantly papules, were present bilaterally, consistent with severe (grade 4) acne on the US Food and Drug Administration (FDA) Investigator’s Global Assessment (IGA) scale for acne severity [4] (Fig. 1). There was evidence of baseline atrophic boxcar scarring. Previous treatment for her acne consisted of a course of topical Duac (benzoyl peroxide and clindamycin) and microdermabrasion, both of which had limited effect. Retinoid therapy was contraindicated due to her family planning considerations.

Three treatments in total of combination non-ablative laser therapy were applied; each session was held 2 to 3 weeks apart. We used a combination of treatment with high fluence potassium titanyl phosphate (KTP) and neodymium-doped: yttrium aluminum garnet (Nd:YAG) laser.

KTP laser settings of 532 nm, high fluence 20 J/cm^2^, 20 millisecond pulse width, 1 mm^2^ spot size, and 2 Hz pulse rate were used superficially, targeting the oxyhemoglobin within the pilosebaceous unit. Nd:YAG laser settings of 1064 nm, 150 to 250 J/cm^2^, 55 to 75 millisecond pulse width, and 1 mm^2^ spot size were used to eradicate the base of a hair follicle on a deeper level of the dermis along with any des-oxyhemoglobin resulting from the more superficial treatment. No topical sensitizer was used. Polarized visual magnification with a 5× headset magnification was used to assist with identification of lesions. External sclera shields were applied to our patient during treatment.

**Fig. 2:**
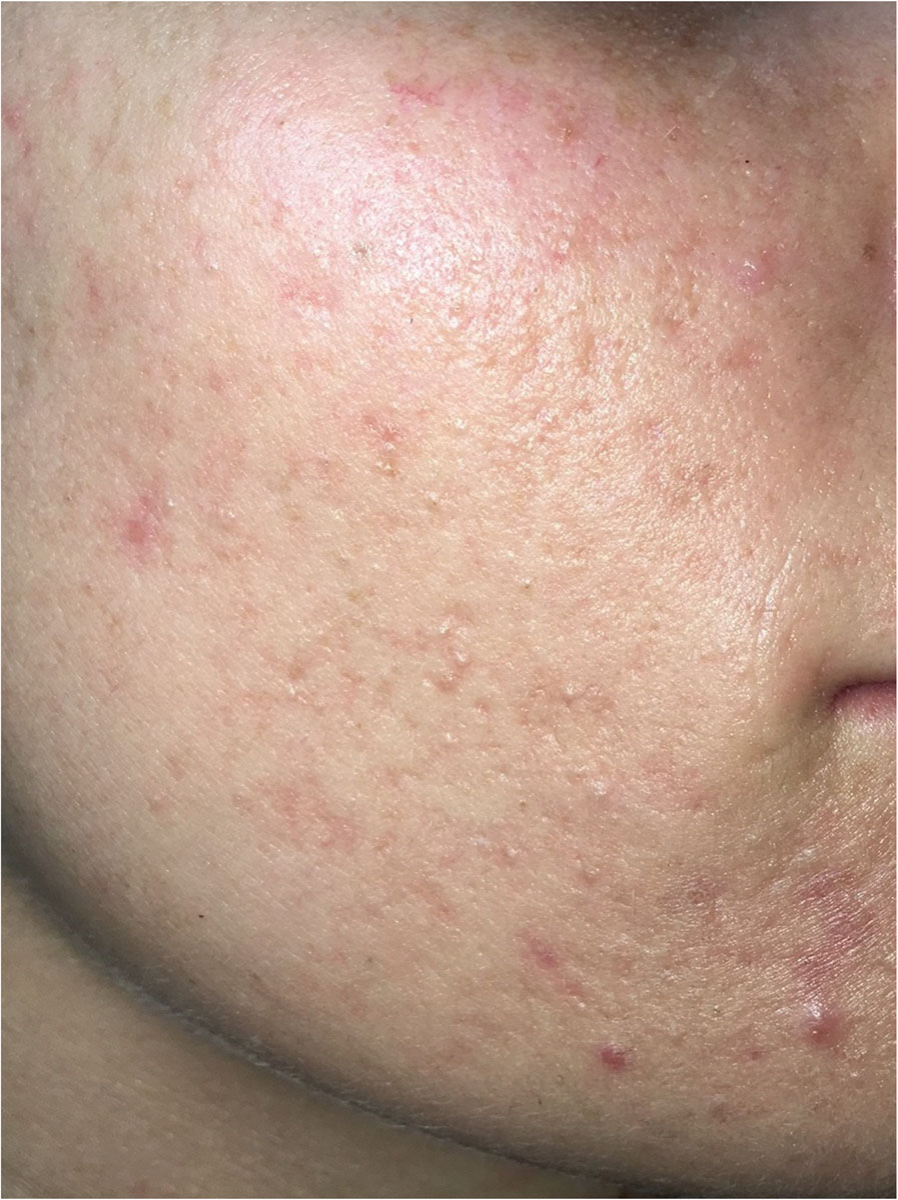
Six months after first treatment

The procedure was painless. On each occasion following treatment, erythema of the treated area was apparent for approximately 3 days but subsided uneventfully with no blistering. There were no late complications. A 73% reduction in total inflammatory lesions (from 37 to 10) was evident on completion of the three applications when our patient was reviewed 6 months after the initial treatment (Fig. 2). This represented a reduction in acne severity from severe (grade 4) to mild (grade 2) on the IGA scale. Photos were taken with iPhone 5 camera 8MP with illumination via a Lifemax High Vision light without flash. Atrophic boxcar scarring, which was evident prior to the treatment, remained. She reported that she was very satisfied with the outcome of the treatment.

## Discussion

The method of phototherapy used in this case aims to precisely target the infected pilosebaceous unit with a narrow spot size green laser using visual augmentation, thus limiting damage to adjacent tissue. An infrared laser is used to target melanin in a deeper layer and resolve the mottled appearance of des-oxyhemoglobin following superficial treatment.

Studies on the use of green light (495 to 570 nm) in the treatment of acne have treated the entire surface of the designated treatment side, or with a notably larger spot size, no visual augmentation, and lower fluence. Baugh and Kucaba demonstrated efficacy with 12 J/cm^2^ fluence treatment of the entire surface of the designated treatment side [5]. Yilmaz *et al*. used 5 to 12 J/cm^2^ fluence with a 4 mm spot size. Neither used magnification aid [6].

Combination laser therapy of different wavelengths of the green and infrared spectrum have been shown to be safe and effective in pulsed dye lasers (PDL) [7] with a postulated synergistic mechanism of action accounting for increased efficacy versus monophasic treatment. A literature search found no comparable studies for the KTP/Nd:YAG combination used in this case.

This case study represents a unique instance of combination KTP laser (532 nm)/Nd:YAG laser (1064 nm) light therapy with visually augmented narrow spot size and high fluence. The outcome of this case indicates that this treatment method is a candidate for further research.

## Conclusions

Optimal parameters in phototherapy for acne are yet to be established. This case illustrates that there may be utility in this novel approach of narrow spot size and magnification-assisted targeted combination laser therapy for inflammatory acne that merits further investigation.
